# Comparison of Multiple Bioactive Constituents in the Flower and the Caulis of *Lonicera japonica* Based on UFLC-QTRAP-MS/MS Combined with Multivariate Statistical Analysis

**DOI:** 10.3390/molecules24101936

**Published:** 2019-05-20

**Authors:** Zhichen Cai, Chengcheng Wang, Lisi Zou, Xunhong Liu, Jiali Chen, Mengxia Tan, Yuqi Mei, Lifang Wei

**Affiliations:** College of Pharmacy, Nanjing University of Chinese Medicine, Nanjing 210023, China; caizhichen2008@126.com (Z.C.); ccw199192@163.com (C.W.); zlstcm@126.com (L.Z.); 18994986833@163.com (J.C.); 18816250751@163.com (M.T.); 18260028173@163.com (Y.M.); weilifang1995@yeah.net (L.W.)

**Keywords:** Lonicerae japonicae flos, Lonicerae japonicae caulis, UFLC-QTRAP-MS/MS, bioactive constituents, multivariate statistical analysis

## Abstract

Lonicerae japonicae flos (LJF) and Lonicerae japonicae caulis (LJC) are derived from different parts of *Lonicera japonica* Thunb. (Caprifoliaceae), and have been used as herbal remedies to treat various diseases for thousands of years with confirmed curative effects. However, little attention has been paid to illustrating the differences in efficacy from the perspective of phytochemistry. In the present study, a simultaneous determination of 47 bioactive constituents, including 12 organic acids, 12 flavonoids, six iridoids, 13 amino acids and four nucleosides in 44 batches of LJF and LJC samples from different habitats and commercial herbs was established based on ultra-fast liquid chromatography tandem triple quadrupole mass spectrometry (UFLC-QTRAP-MS/MS). Moreover, principal component analysis (PCA), partial least squares discriminant analysis (PLS-DA) and *t*-test were then performed to classify and reveal the differential compositions of LJF and LJC according to the content of the tested constituents. The results demonstrated that the types and contents of chemical components (e.g., isochlorogenic acid A, chlorogenic acid, neochlorogenic acid, quinic acid, secologanic acid, luteoloside, loganin, secoxyloganin, morroniside and L-isoleucine) were significantly different, which may lead to the classification and the differences in efficacy of LJF and LJC. Our findings not only provide a basis for the comprehensive evaluation and intrinsic quality control of LJF and LJC, but also pave the way for discovering the material basis contributing to the different properties and efficacies of the two medicinal materials at the phytochemical level.

## 1. Introduction

It is a common procedure to obtain two or more herbal materials from different parts of the same medicinal plant. Lonicera Lonicerae japonicae flos (LJF) and Lonicera Lonicerae japonicae caulis (LJC) are typical representatives of this. They are derived from different parts of *Lonicera japonica* Thunb., which belongs to the Caprifoliaceae family. LJF is originated from the dried buds and flowers, while LJC is derived from the dried stems. They are commonly referred to as “jin-yin-hua” and “ren-dong-teng” respectively, as independent Chinese material medica in the Chinese Pharmacopoeia (2015 edition, Volume I) and possess various pharmacological actions, such as anti-inflammatory [1,2,3,4], anti-oxidant [5], hepatoprotective [6], anti-bacterial [7], anti-viral [8], anti-tumor [9], immunity enhancement [10] and other biological activities.

In the past decades, the great majority of literature about *L*. *japonica* has focused on identifying chemical compounds, quality control, and pharmacological action [11,12,13,14,15]. More than 200 compounds such as flavonoids, organic acid, iridoids, saponins and volatile oil have been identified in *L*. *japonica*, and these active ingredients greatly contribute to its excellent function in the clinic [16,17,18,19]. Phenolic acids, especially chlorogenic acid and caffeic acid, are regarded as the main anti-inflammatory active ingredients. Chlorogenic acid also has excellent anti-tumor, anti-oxidant and hepatoprotective activities. The activities of flavonoids include anti-oxidant, anti-bacterial and anti-tumor ones. A large number of studies also indicated that protocatechuic acid, chlorogenic acid and luteolin have anti-tumor activities [20,21]; loganin and morroniside have neuroprotective [22,23], anti-thrombotic and anti-coagulant effects. Loganin and sweroside exhibit analgesic and anti-inflammatory activities [24]; Inosine may prove to be beneficial in the treatment of rheumatic heart disease, acute and chronic hepatitis. Although the efficacy of LJF and LJC described in the current Chinese Pharmacopoeia is different, the reason for these differences remains unclear. At present, more attention is paid to the quality control and chemical composition with less attention to the relation to traditional efficacy, resulting in the lack of material basis research relating chemical compositions and traditional efficacies.

The determination of single or several bioactive compounds in herbal medicines is one-sided, which might incompletely represent its intrinsic quality because the wholeness of traditional Chinese medicines (TCMs) means multi-components at multi-targets. Then, the development of hyphenated chromatography technique makes the simultaneous analysis of numerous constituents in short time possible [25,26,27]. Hence, the aim of this study is to explore the difference of synthesis and accumulation of metabolites in the flowers and the caulis of *L. japonica* based on simultaneous determination of multiple bioactive compounds combined with multivariate statistical analysis. A reliable and accurate method based on the ultra-fast liquid chromatography tandem triple quadrupole mass spectrometry (UFLC-QTRAP-MS/MS) have been developed for the simultaneous determination of 47 bioactive constituents, including 12 organic acids, 12 flavonoids, six iridoids,13 amino acids and four nucleosides in 44 batches of LJF and LJC. In addition, principal component analysis (PCA), partial least squares discriminant analysis (PLS-DA) and *t*-test have been performed to classify the samples and reveal the differential compositions between LJF and LJC according to the contents of the tested constituents. Our findings not only provide a basis for the comprehensive evaluation and intrinsic quality control of LJF and LJC, but also pave the way for discovering the material basis contributing to the different properties and efficacies of the two medicinal materials at the phytochemical level.

## 2. Results 

### 2.1. Optimization of Extraction Conditions

In order to achieve the optimal extraction conditions, three factors combining the previous experiences with the nature of the components had been chosen to be investigated and optimized. three parameters setting as follows: extraction solvents (100% methanol, 70% methanol, and 25% methanol), solvent to sample ratios (20:1, 40:1, and 60:1 (*v/w*)) and extraction time (20 min, 40 min, and 60 min), which might have both positive and negative effects on the extraction efficiency. The results revealed that the extraction efficiency of 70% methanol was similar to that of 100% methanol. With comprehensive consideration of the properties of the detected compounds, 70% methanol was chosen as extraction solvents. Moreover, ultrasonic extraction with solvent to sample ratio 40:1 for 45 min was sufficient and appropriate for the analysis. Therefore, the optimal extraction conditions were ultrasonic extraction with a 40:1 ratio of 70% methanol for 45 min at room temperature.

### 2.2. Optimization of UFLC Conditions

Three types of columns: (X Bridge R C_18_ (4.6 mm × 100 mm, 3.5 μm) (Waters, Wexford, Ireland), Agilent ZORBAX SB C_18_ column (250 mm × 4.6 mm, 5 μm) (Agilent, Palo Alto, CA, USA)and Thermo Acclaim TM RSLC 120 C_18_ (150 mm × 2.1 mm, 2.2 μm) (Thermo Scientific, Waltham, MA, USA), different mobile phases (water/acetonitrile, water/methanol, 0.1% aqueous formic acid/ acetonitrile, 0.2% aqueous formic acid/0.2% formic acid acetonitrile), different flow rates (0.3 mL/min, 0.8 mL/min, 1.0 mL/min), and column temperatures (25 °C, 30 °C, and 35 °C) were all compared to test samples. The results of UFLC showed that the column of X Bridge R C_18_ (4.6 mm × 100 mm, 3.5 μm) (Waters, Wexford, Ireland) was better because of the strong hydrophilicity of organic acids, amino acids and nucleosides. Water-acetonitrile system had better resolution than water-methanol system. And when the mobile phase was added with formic acid, the shape and symmetry of chromatographic peak of organic acids were significantly improved. Finally, 0.2% aqueous formic acid-0.2% formic acid acetonitrile at a flow rate of 0.8 mL/min under the column temperature of 30 °C was selected and applied.

### 2.3. Optimization of MS Conditions

The individual solutions of all standard compounds (about 100 ng/mL) were examined with the electro spray ionization (ESI) source in the positive and negative ion modes. The most abundant fragment ions were chosen as MRM transition from MS/MS spectrum; All the optimum values including retention time (t_R_), precursor and product ions, De-clustered Voltage (DP) and collision energy (CE) of each compound are summarized in Table 1 and the chromatograms with MRM mode are presented in Figure S1. After trial and error inspection, we found that kaempferol-3-O-rutinoside, amino acids and nucleosides had higher sensitivity and intensity in positive ion mode. As shown in Table 1, chlorogenic acid, neochlorogenic acid and cryptochlorogenic acid as isomer; isochlorogenic acid A, isochlorogenic acid B, isochlorogenic acid C and 1,3-*O*-dicaffeoylquinic acid as isomer; hyperoside, isoquercitrin as isomer; luteoloside, astragalin as isomer; L-leucine, L-isoleucine as isomer; lonicerin, kaempferol-3-*O*-rutinoside as isomer; they have the same precursor ion-product ion pairs, respectively. Thus, those standard substances were sequentially injected into QTRAP-MS/MS in turn to determine the compound according to the retention time.

### 2.4. Analytical Method Validation

The detail of validation results of the method were presented in Table 2. The standard calibration curves showed good determination coefficients (*r* > 0.9990) of all the analytes. The limits of detections (LODs) and limits of quantifications (LOQs) ranged from 0.0050–139.226 ng/mL and 0.015–417.797 ng/mL, respectively. The RSD values of intra-day, inter-day of the 47 analytes ranged from 0.58–4.28% and 0.63–4.68%, respectively. The values of repeatability, stability test of the 47 components were all less than 5%, and the mean recoveries varied between 93.89% and 104.13%, with the RSD% less than 4.88%, which verified the effectiveness of the proposed method.

### 2.5. Quantitative Analysis of Samples

**Figure 1:**
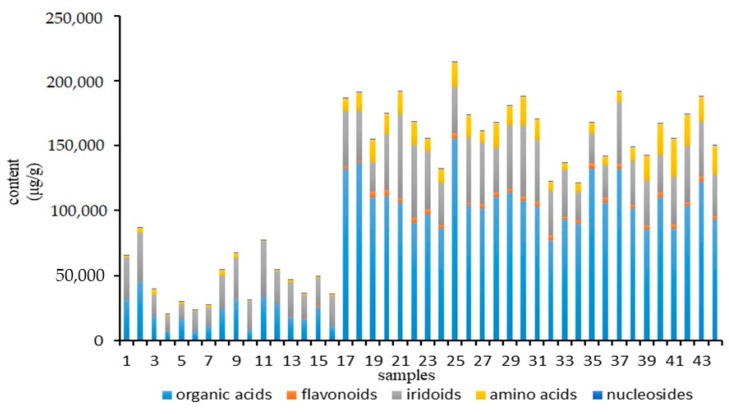
Histogram of the accumulative contents of LJF and LJC from 44 batches (*X*-axis represents 44 batches of samples; *Y*-axis is the content (μg/g) of five types of compounds in each sample).

The sample information is listed in Table 3. The validated analytical method was employed to assay 47 analytes in 44 batches of samples. The results of contents of 47 analytes are summarized in Table S1. The histogram (Figure 1) illustrated that the contents of 47 analytes in LJF were higher than LJC apparently. And organic acids were the most abundant constituents among LJF. However, the percentages of the content of iridoids were bigger in LJC than LJF. From the results of the test data, we can know that the contents of isochlorogenic acid A, chlorogenic acid, neochlorogenic acid, quinic acid, luteoloside, luteolin and L-proline were relatively higher in LJF. Regardless of the organic acids or flavonoids, the measured contents of LJF are all exceeding the ones in LJC. Furthermore, loganin has significantly high content of LJC. therefore, it is considered as a characteristic component of LJC in current Chinese Pharmacopoeia. By comparing these parameters, it could be found that the contents and constituents of LJF and LJC were quite different. 

### 2.6. PCA of Samples

**Figure 2:**
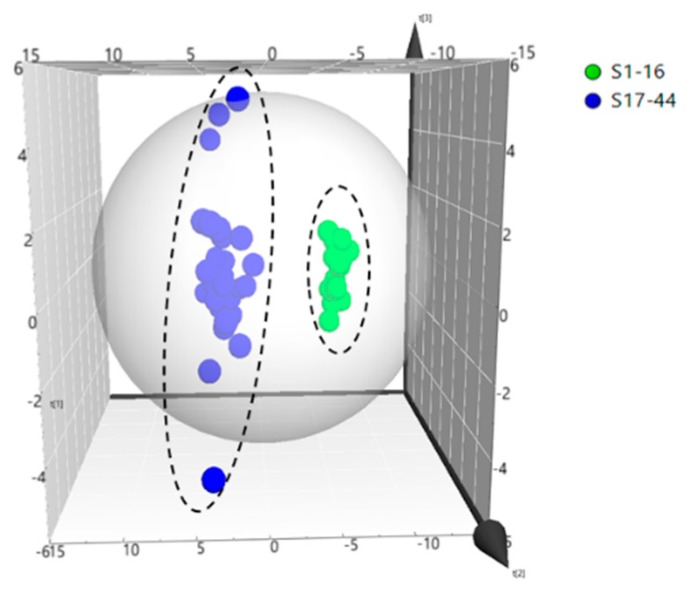
Score scatter plot by PCA processed data acquired from samples. Each of the blue circle represented a batch of LJF, the green circle represented a batch of LJC.

Unsupervised principal component analysis (PCA) was performed to distinguish and assess the quality of LJF and LJC. The measured contents (μg/g) of 47 compounds were set as variables. The scores plot was displayed in Figure 2., which showed a clear variation in two principal components (PC1, PC2). In terms of PCA analysis, the samples were divided into two clusters (LJF and LJC), which indicated that the composition and contents of LJF were quite different from LJC. The two principal components accumulatively accounted for 84%, This showed that the two principal components can fully reflect the overall information.

### 2.7. PLS-DA of the Samples

**Figure 3:**
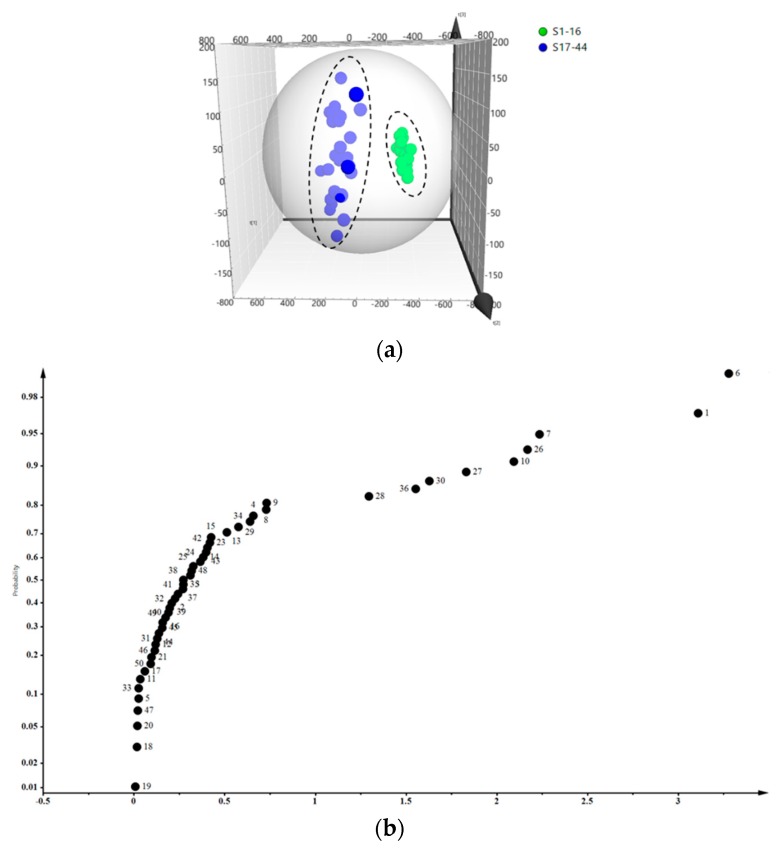
Score scatter plot (**a**) and VIP (**b**) by PLS-DA processed data obtained from LJF and LJC. Each of the blue circle represented a batch of LJF, the green circle represented a batch of LJC, each of the black circle represented a compound (see Table 1 for compound names).

In order to find the potential chemical markers that had a significant impact on sample discrimination, the partial least squares discriminant analysis (PLS-DA) and variable importance in the projection (VIP) tests were performed. The PLS-DA scores plot and VIP values are shown in Figure 3a,b. LJF and LJC also were separated into two groups. Thereby indicated the remarkable differences of chemical constitutes between LJF and LJC. The specific parameters set as follows: confidence level was 95%, R2Y = 0.962 and Q2 = 0.935.

VIP indicates the importance of variables to the model. It describes the overall contribution of each variable to the model and the threshold is usually set to VIP > 1. In this experiment, the VIP-values were obtained from PLS-DA processed data. Among them, VIP > 1 indicates important variables, which could be regarded as potential markers that contribute greatly to the classification of these samples, such as isochlorogenic acid A (**1**), chlorogenic acid (**6**), neochlorogenic acid (**7**), quinic acid (**10**), secologanic acid (**26**), loganin (**27**), secoxyloganin (**28**), morroniside (**30**) and L-isoleucine (**36**). 

### 2.8. t-test of Samples

**Figure 4:**
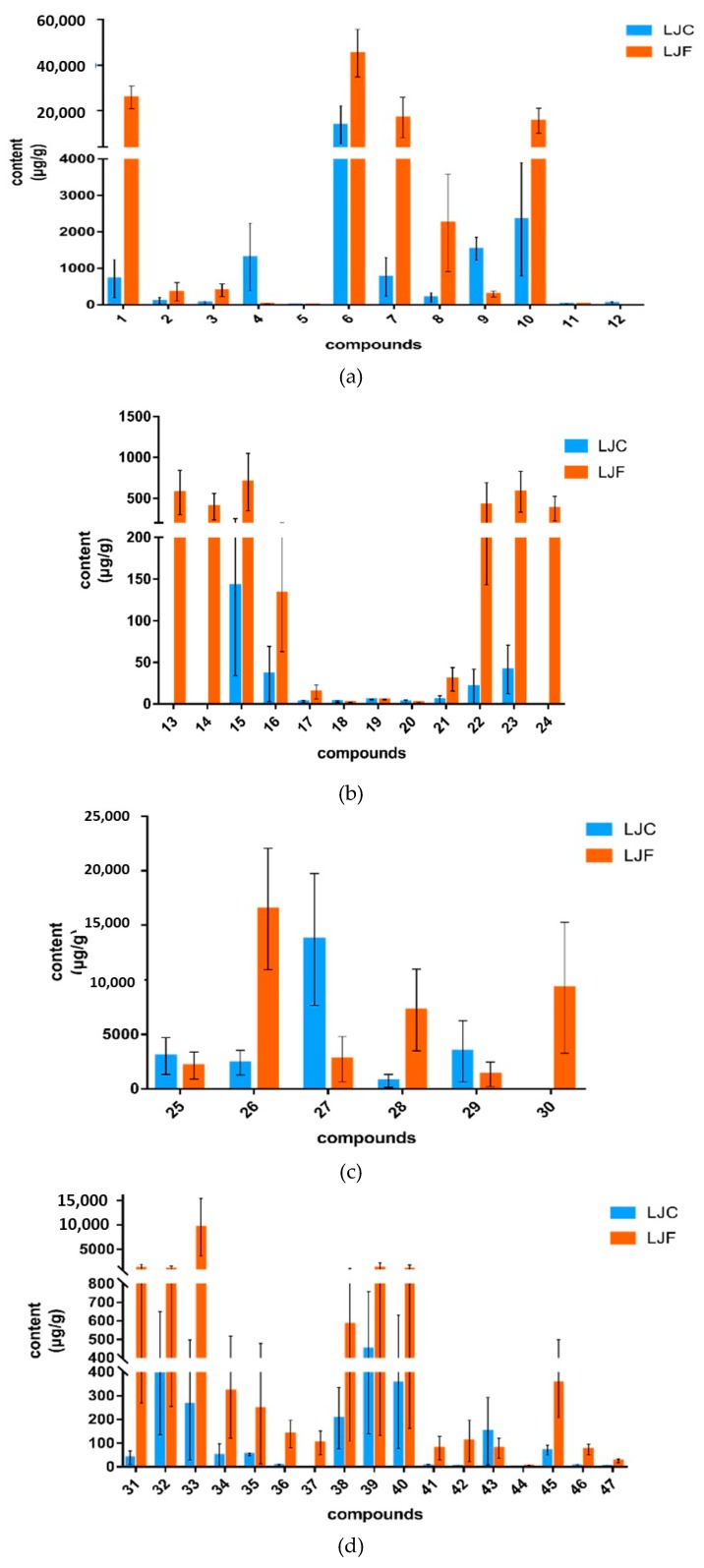
The contents of 47 compounds in samples. (**a**) organic acids, (**b**) flavonoids, (**c**) iridoids, (**d**) amino acids and nucleosides (*X*-axis 1–47 is the number of compounds, see Table 1 for compound names; *Y*-axis is the content (μg of compound/g)).

A *t*-test was used to verify the occurring probability of the differences. According to the *t*-test (Figure 4), the quantitative constituents in this experiment revealed a remarkable difference between LJF and LJC, except for protocatechuic acid, apigenin, sweroside, L-serine. The content of isochlorogenic acid A, neochlorogenic acid, quinic acid, rutin, hyperoside, luteoloside, lonicerin, kaempferol-3-O-rutinoside, isoquercitrin, secologanic acid, secoxyloganin and L-proline had strikingly higher level (*p* < 0.01) in LJF compared with LJC. While, quantitation of loganin, 1,3-*O*-dicaffeoylquinic acid, sweroside and loganin acid displayed super high level (*p* < 0.01) in LJC. Luteoloside and loganin were identified as the most effective chemical markers to evaluate the quality of LJF and LJC respectively, which is the same as the record of Chinese Pharmacopoeia.

## 3. Discussion

In this study, a sensitive and efficient method was developed and validated to explore the difference of synthesis and accumulation of metabolites in the flower and the caulis of *L*. japonica based on simultaneous determination of multiple bioactive constituents combined with multivariate statistical analysis. The results of PCA, PLS-DA and *t*-test were almost consistent, indicating that UFLC-QTRAP-MS/MS method combined with multivariate statistical analysis might be successful for quality evaluation of TCMs. In this method, precursor and product ion monitoring not only increase the specificity of detection but also help to identify the molecules. This can overcome the deficiencies of traditional methods and effectively reveal the complexity of samples ingredients.

From Figure 3b, the VIP-value of compounds **7**, **10**, **26**, **27**, **28**, **30**, **36** is close, with a range of 1.25–2.5. While, for compounds **1** and **6** VIP is > 3. Isochlorogenic acid A (**1**) and chlorogenic acid (**6**) are phenolic acids. It is widely recognized that phenolic acids is the primary active components, and among them, chlorogenic acid is the major ingredient for a wide range of pharmacological activities [2,3,4,28,29] and its content has been used as the main indicator for evaluating the quality of LJF; While, in the Chinese Pharmacopoeia chlorogenic acid also recorded as a marker of LJC. This might be the material basis of their common efficacy of “qing-re-jie-du”. However, the standard of content in LJC is 15 times lower than that of LJF. And the PLS-DA and VIP results of indicated the chlorogenic acid could be considered as a potential marker distinguish between LJF and LJC, which is consistent with the Chinese Pharmacopoeia record. Generally, different compounds and different types of compounds have different pharmacological effects. The results of quantitative analysis may provide the support for elucidating the similarities and differences in the efficacy of LJF and LJC from the perspective of phytochemistry.

## 4. Materials and Methods

### 4.1. Plant Materials

The forty-four sample batches were from different habitats and commercial herbs. Detailed information on these samples is listed in Table 3. The botanical origins of materials were authenticated by one of the authors, Prof. Xunhong Liu. Voucher specimens were deposited at the Herbarium in Nanjing University of Chinese Medicine. The samples were collected in 2018, samples 1–16 are Lonicerae japonicae caulis, and samples 17–44 are Lonicerae japonicae flos. 

### 4.2. Chemicals and Reagents

Forty-seven chemical standards were obtained: isochlorogenic acid A (**1**), isochlorogenic acid B (**2**), isochlorogenic acid C (**3**), 1,3-*O*-dicaffeoylquinic acid (**4**), 4,5-*O*-dicaffeoylquinic acid methyl ester (**5**), chlorogenic acid (**6**), neochlorogenic acid (**7**), cryptochlorogenic acid (**8**), caffeic acid (**9**), quinic acid (**10**), protocatechuic acid (**11**), ferulic acid (**12**), rutin (**13**), hyperoside (**14**), luteoloside (**15**), luteolin (**16**), rhoifolin (**17**), diosmetin (**18**), apigenin (**19**), kaempferol (**20**), astragalin (**21**), lonicerin (**22**), kaempferol-3-*O*-rutinoside (**23**), isoquercitrin (**24**), sweroside (**25**), secologanic acid (**26**), loganin (**27**), secoxyloganin (28), loganin acid (**29**), morroniside (**30**), L-alanine (**31**), L-serine (**32**), L-proline (**33**), L-valine (**34**), L-threonine (**35**), L-isoleucine (**36**), L-leucine (**37**), L-aspartic acid (**38**), L-glutamate (**39**), L-lysine (**40**), L-histidine (**41**), L-phenylalanine (**42**), L-arginine (**43**), cytidine (**44**), uridine (**45**), adenosine (**46**) and inosine (**47**). The purity of all standards components was determined to be more than or equal to 98% by HPLC analysis. The structure of the standard substance is showed in Figure S2. Among them **1**, **2**, **6**–**9**, **12**, **27** and **31**-**47** were obtained from Shanghai Yuanye Biotechnology Co. Ltd. (Shanghai, China); **3**, **4**, and **11** were received from Chengdu Prefa Technology Development Co. Ltd. (Sichuan, China); **5**, **15**–**17**, **22**, **26** and **29** were provided by Liangwei Chemical Reagent Co. Ltd. (Nanjing, China); **10**, **13**, **14**, **21** and **24** were purchased from the Department of Control of Pharmaceutical and Biological Products (Beijing, China); **18**–**20**, **23**, **25** and **30** were offered by Chengdu Chroma Biotechnology Co. Ltd. (Sichuan, China); **28** was acquired from Nanjing Jingzhu Biotechnology Co. Ltd. (Nanjing, China). Chromatographic grade methanol and acetonitrile were purchased from Merck (Darmstadt, Germany); other analytical grade solvents were purchased from Liangwei Chemical Reagent Co. Ltd. (Nanjing, China). Ultrapure water was obtained from a Milli-Q purification system (Millipore, Bedford, MA, USA).

### 4.3. Preparation of Sample Solutions

The samples were ground into powder (50 meshes). Accurately weighed powder samples (1.0 g) were extracted by ultra-sonication in 40 mL 70% methanol for 45 min, then cooled to room temperature; The extraction solution was allowed to be cooled and weighed again, the loss of solvent was replenished with 70% methanol and mixed well. After centrifugation (8050 g, 10 min) and filtered (0.22 μm membrane filters), the supernatants were stored in a sample bottle at 4 °C prior to injection LC-MS.

### 4.4. Preparation of Standard Solutions 

The following amounts of each sample in mg/mL were used to prepare standard solutions: (**1**) 10.06, (**2**) 1.09, (**3**) 1.19, (**4**) 1.18, (**5**) 1.09, (**6**) 10.09, (**7**) 10.06, (**8**) 5.05, (**9**) 1.22, (**10**) 10.01, (**11**) 1.12, (**12**) 0.96, (**13**) 1.26, (**14**) 1.33, (**15**) 1.16, (**16**) 5.09, (**17**) 1.42, (**18**) 1.09, (**19**) 4.93, (**20**) 1.08, (**21**) 0.99, (**22**) 1.09, (**23**) 1.02, (**24**) 1.06, (**25**) 5.08, (**26**) 5.04, (**27**) 5.08, (**28**) 5.07, (**29**) 5.07, (**30**) 5.10, (**31**) 5.09, (**32**) 5.09, (**33**) 5.09, (**34**) 1.02, (**35**) 1.06, (**36**) 0.83, (**37**) 1.00, (**38**) 1.02, (**39**) 0.76, (**40**) 0.57, (**41**) 1.10, (**42**) 1.08, (**43**) 0.88, (**44**) 1.25, (**45**) 1.02, (**46**) 1.12, (**47**) 1.15. All solutions were stored at 4 °C, then filtered through 0.22 μm membranes (Jinteng Laboratory Equipment, Tianjin, China) before analysis.

### 4.5. UFLC-QTRAP-MS/MS Instrumentation and Conditions

All samples were analyzed using UFLC system (Shimadzu Corp., Kyoto, Japan) coupled with a triple quadrupole-linear ion trap mass spectrometer (QTRAP-5500) (AB SCIEX, Framingham, MA, USA). The ESI-MS spectra were acquired in the multiple reaction monitoring (MRM) under both positive and negative ion modes. The MS parameters were setting as follows: gas temperature 550 °C; the ion spray voltage was set to 5500 V (positive) and −4500 V (negative), respectively; gas temperature 550 °C; GS1 flow 55 L/min; GS2 flow 55 L/min; CUR flow 40 L/min; all MS data were acquired and analyzed using the Analyst 1.6.2 software. The cone voltage and collision energy parameter of each compound was individually optimized. Separation was performed using the X Bridge R C_18_ column (4.6 mm × 100 mm, 3.5 μm) (Waters, Wexford, Ireland). The mobile phase was composed of 0.2% aqueous formic acid (A) and acetonitrile with 0.2% formic acid (B) at the flow rate of 0.8 mL/min. The injection volume was 1 μL. The gradient elution as follows: 0–5 min: 2% B; 5–10 min: 2–13% B; 10–12 min: 13% B; 12–17 min: 13–25% B; 17–25 min: 25–33% B; 25–27 min: 33–35% B; 27–29 min: 35–50% B; 29–31 min: 50–95% B. The re-equilibration time was 4 min.

### 4.6. Validation of UFLC-QTRAP-MS/MS Method

The analysis method was validated for linearity and range, detection limit (LOD), limit of quantitation (LOQ), precision (intra-day and inter-day), repeatability, stability and accuracy according to the guidelines of the International Conference on Harmonisation of Technical Requirements for Registration of Pharmaceuticals for Human Use (ICH) Q2 analytical validation. The mixed standard stocked solution containing forty -four reference substances was serially diluted with 70% methanol to required concentrations for the establishment of calibration curves. The LODs and LOQs of these analytes under the present chromatographic conditions were determined at signal-to-noise (S/N) ratio equaled to 3 and 10, respectively. The precision of the method was evaluated by determining the 47 analytes in six replications during one day and by duplicating the experiments on three consecutive days. Six independent sample solutions from the same sample were parallelly processed and analyzed to ensure the repeatability. The same sample solution was injected at 0, 2, 4, 8, 12 and 24 h to investigate the stability of the instrument, respectively. A recovery test was used to check the accuracy of the method. Recovery values were calculated by the formula: (%) = (found amount – original amount in sample)/spiked amount × 100%.

### 4.7. Multivarite Statistical Analysis

In order to observe the classification and assess the variations of LJF and LJC, the data of 47 analytes were used to carry out PCA and PLS-DA using the SIMCA-P 13.0 software (Umetrics AB, Umea, Sweden). PCA is an unsupervised pattern recognition method, which is used to visualize similarities or differences in multivariate data. It has been widely used in the differentiation and identification of medicinal materials. PLS-DA was excellent for highlighting the differences between two groups. *t*-test is utilized to confirm that there were differences in chemical composition between LJF and LJC (SPSS 16.0 for Windows, IBM, Armonk, NY, USA). Data of the contents of 47 compounds in the 44 batches of samples are listed in Table S1. When the contents of investigated components were below the quantitation limit or not detected in the samples, the values of such elements were considered to be 0.

## 5. Conclusions

In the present study, an optimized UFLC-QTRAP-MS/MS assay has been successfully applied to simultaneous determination of 47 bioactive constituents, including 12 organic acids, 12 flavonoids, six iridoids, 13 amino acids and four nucleosides in 44 batches of LJF and LJC samples from different habitats and commercial herbs. Furthermore, PCA, PLS-DA and *t*-test were performed to classify the samples and reveal the differential compositions between LJF and LJC according to the content of the tested constituents. The results showed that LJF and LJC were clearly classified and the sample classification were closely related to the different chemical constituents. (e.g., isochlorogenic acid A, chlorogenic acid, neochlorogenic acid, quinic acid, secologanic acid, luteoloside, loganin, secoxyloganin, morroniside and L-isoleucine). It demonstrated that the quantitative components in this experiment had remarkable differences between LJF and LJC based on *t*-test, except for protocatechuic acid, apigenin, sweroside and L-serine. The bioactivities of loganin, including neuroprotective [20,21], anti-thrombotic and anti-coagulant effects, are consistent with the efficacy of loganin (the biomarker of LJC) recorded in the Chinese Pharmacopoeia. The content determination also illustrated that loganin in LJC accounted for a significantly higher percentage than that in LJF, while LJF was more abundant in luteoloside, leading to great influences on the different efficacy that LJF and LJC were separately better in “shu-san-feng-re” and “shu-feng-tong-luo”. LJF and LJC are medicinal materials with different medicinal parts of the same origin. Although the types of chemical components are basically the same, their efficacy are different. Our findings not only provided a basis for the comprehensive evaluation and intrinsic quality control of LJF and LJC, but also paves the way for discovering the material basis contributing to the different properties and efficacies of the two medicinal materials at the phytochemical level.

## Figures and Tables

**Table 1:** Retention times and related mass spectrometry (MS) data of the target compounds.

No.	Name	Formula	(t_R_) (min)	[M + H]^+^ m/z	[M − H]^−^ m/z	MRM (Precursor→Product)	DP/V	CE/V
1	Isochlorogenic acid A	C_25_H_24_O_12_	20.15	-	515.45	515.1/191	−85	−22
2	Isochlorogenic acid B	C_25_H_24_O_12_	20.13	-	515.45	514.989/353	−80	−26
3	Isochlorogenic acid C	C_25_H_24_O_12_	20.27	-	515.45	515.1/191	−75	−24
4	1,3-*O*-dicaffeoylquinic acid	C_25_H_24_O_12_	20.30	-	515.45	514.980/190.979	−95	−24
5	4,5-*O*-dicaffeoylquinic acid methyl ester	C_26_H_26_O_12_	29.50	-	529.47	529.194/135.001	−85	−42
6	Chlorogenic acid	C_16_H_18_O_9_	18.75	-	353.31	305.01/125	−35	−20
7	Neochlorogenic acid	C_16_H_18_O_9_	17.64	-	353.31	305.01/125	−80	−26
8	Cryptochlorogenic acid	C_16_H_18_O_9_	19.86	-	353.31	305.01/125	−95	−20
9	Caffeic acid	C_9_H_8_O_4_	19.45	-	179.16	179.03/134.6	−125	−20
10	Quinic acid	C_7_H_12_O_6_	18.75	-	191.17	191.099/84.981	−195	−28
11	Protocatechuic acid	C_7_H_6_O_4_	12.99	-	153.12	152.9/109	−85	−16
12	Ferulic acid	C_10_H_10_O_4_	23.89	-	193.18	193.017/134	−50	−10
13	Rutin	C_27_H_30_O_16_	22.06	-	609.52	609.06/300	−245	−46
14	Hyperoside	C_21_H_20_O_12_	22.74	-	463.38	463.003/299.9	−160	−36
15	Luteoloside	C_21_H_20_O_11_	23.03	-	447.38	447.117/284.963	−300	−36
16	Luteolin	C_15_H_10_O_6_	29.53	-	285.24	285.086/132.980	−170	−40
17	Rhoifolin	C_27_H_30_O_14_	24.82	-	577.52	577.185/268.958	−65	−46
18	Diosmetin	C_16_H_12_O_6_	30.88	-	299.26	298.938/283.929	−215	−30
19	Apigenin	C_15_H_10_O_5_	30.74	-	269.24	268.8/116.9	−129	−40
20	Kaempferol	C_15_H_10_O_6_	30.88	-	285.24	285.0/116.9	−120	−36
21	Astragalin	C_21_H_20_O_11_	24.40	-	447.38	447.1/283.9	−100	−36
22	Lonicerin	C_27_H_30_O_15_	23	-	593.52	593.146/283.984	−200	−54
23	Kaempferol-3-O-rutinoside	C_27_H_30_O_15_	23.59	595.5	-	595/287.2	36	25
24	Isoquercitrin	C_21_H_20_O_12_	22.50	-	464.38	463.015/300	−180	−36
25	Sweroside	C_16_H_22_O_9_	20.14	-	358.34	357.213/124.985	−65	−20
26	Secologanic acid	C_16_H_22_O_10_	19.17	-	376.36	357.107/212.956	−170	−22
27	Loganin	C_17_H_26_O_10_	19.72	-	390.38	389.262/226.980	−40	−12
28	Secoxyloganin	C_17_H_24_O_11_	21.10	-	404.37	403.219/120.973	−135	−32
29	Loganin acid	C_16_H_24_O_10_	18.07	-	376.36	375.107/212.956	−170	−22
30	Morroniside	C_17_H_26_O_11_	18.53	-	406.38	405.235/243	−100	−14
31	l-Alanine	C_3_H_7_NO_2_	1.38	90.09	-	90.06/44.02	100	10
32	l-Serine	C_3_H_7_NO_3_	1.38	106.09	-	106.05/59.99	100	8
33	l-Proline	C_5_H_9_NO_2_	1.65	116.13	-	116.07/70.02	68	10
34	l-valine	C_5_H_11_NO_2_	2.32	118.15	-	118.09/72.06	100	10
35	l-Threonine	C_4_H_9_NO_3_	1.38	120.12	-	120.07/74	100	20
36	l-Isoleucine	C_6_H_13_NO_2_	4.96	132.17	-	132.1/86.05	64	10
37	l-Leucine	C_6_H_13_NO_2_	5.40	132.17	-	132.1/86.05	100	16
38	l-aspartic acid	C_4_H_7_NO_4_	1.38	134.10	-	134.05/87.96	59	10
39	l-Glutamate	C_5_H_7_NO_4_	1.24	146.11	-	147.08/83.92	100	16
40	l-lysine	C_6_H_14_N_2_O_2_	1.25	147.19	-	147.11/83.91	100	14
41	l-Histidine	C_6_H_9_N_3_O_2_	1.24	156.15	-	156.08/110.03	100	16
42	l-Phenylalanine	C_9_H_11_NO_2_	13	166.19	-	166.1/120.05	100	14
43	l-Arginine	C_6_H_14_N_4_O_2_	1.36	175.20	-	175.12/70.02	100	18
44	Cytidine	C_9_H_13_N_3_O_5_	1.65	244.22	-	244.09/112	61	10
45	Uridine	C_9_H_12_N_2_O_6_	4.25	245.20	-	244.896/113	10	13
46	Adenosine	C_10_H_13_N_5_O_4_	6.73	268.24	-	268.1/136.07	86	23
47	Inosine	C_10_H_12_N_4_O_5_	9.62	269.22	-	269/137.07	46	15

**Table 2:** Regression equations, limits of detections (LODs), limits of quantifications (LOQs), intra- and inter-day precisions, repeatability, stabilities, and recoveries of 47 compounds.

Name	Regression Equation	*r*	Linear Range (ng/mL)	LOD (ng/mL)	LOQ (ng/mL)	Precision		Repeatability	Stability	Recovery	
						Intra-Day (RSD%; *n* = 6)	Inter-Day (RSD%; *n* = 3)	(RSD %; *n* = 6)	(RSD%; *n* = 6)	Mean	RSD%
Isochlorogenic acid A	y = 1.02 × 10^3^x + 1.41 × 10^5^	0.9996	77.6–388,000	14.48	43.45	2.63	2.79	3.04	4.92	96.11	3.11
Isochlorogenic acid B	y = 602x + 1.45 × 10^5^	0.9995	7.63–38,200	2.10	6.28	3.95	4.39	0.59	1.12	96.07	4.11
Isochlorogenic acid C	y = 483x + 2.1 × 10^4^	0.9998	45–28,100	7.92	23.76	3.27	3.64	2.79	1.79	99.05	2.02
1,3-*O*-dicaffeoylquinic acid	y = 5.78 × 10^3^x + 5.62 × 10^4^	0.9996	42–2630	6.97	20.91	3.44	3.28	2.49	2.07	98.95	4.75
4,5-*O*-dicaffeoylquinic acid methyl ester	y = 9.67 × 10^3^x − 4.3 × 10^4^	0.9996	5.6–700	1.32	3.96	3.79	4.10	1.01	1.36	94.57	3.98
Chlorogenic acid	y = 845x + 4.77 × 10^5^	0.9999	0.604–755,000	0.20	0.59	1.78	1.79	0.70	0.51	99.48	0.62
Neochlorogenic acid	y = 115x + 1.13 × 10^5^	0.9992	3.01–376,000	0.39	1.16	1.83	1.71	1.77	4.55	98.25	1.70
Cryptochlorogenic acid	y = 878x + 6.7 × 10^3^	0.9993	0.316–79,000	0.09	0.27	4.04	4.31	2.41	4.75	96.61	3.62
Caffeic acid	y = 2.49 × 10^3^x + 1.69 × 10^5^	0.9998	5.76–7200	1.17	3.51	2.86	3.17	3.19	4.91	95.76	3.05
Quinic acid	y = 1.19 × 10^3^x + 3.05 × 10^5^	0.9996	19.6–245,000	5.13	15.39	2.73	2.38	2.26	1.74	96.52	3.68
Protocatechuic acid	y = 6.13 × 10^3^x + 1.95 × 10^5^	0.9994	7.68–960	0.78	2.34	2.50	2.30	1.46	3.31	96.71	4.02
Ferulic acid	y = 121x + 3.68 × 10^3^	0.9990	11.2–1400	2.81	8.44	4.21	3.96	0.87	2.57	102.08	4.88
Rutin	y = 2.5 × 10^3^x + 3.14 × 10^5^	0.9998	0.979–12,200	0.04	0.11	2.91	3.21	1.70	1.00	97.17	2.94
Hyperoside	y = 4.32 × 10^3^x + 1.77 × 10^5^	0.9995	0.265–3313	0.06	0.17	1.69	1.55	4.12	2.50	96.67	4.07
Luteoloside	y = 1.07 × 10^3^x + 1.34 × 10^5^	1.000	0.664–41,500	0.06	0.17	1.03	0.92	4.91	4.01	104.13	4.73
Luteolin	y = 100x + 2.47 × 10^3^	0.9995	124–15,600	10.15	30.45	2.28	2.22	3.13	1.89	101.81	3.68
Rhoifolin	y = 1.08 × 10^4^x + 572	0.9999	1.3–162	0.28	0.84	2.53	2.75	3.08	4.44	99.63	4.74
Diosmetin	y = 1.15 × 10^4^x − 9.93 × 10^3^	0.9993	1.72–214	0.15	0.44	1.09	0.78	0.84	0.47	95.63	1.14
Apigenin	y = 2.17 × 10^4^x − 9.13 × 10^4^	0.9995	25.4–318	4.19	12.56	3.54	3.83	4.22	4.39	99.03	3.61
Kaempferol	y = 191x + 250	0.9997	2.02–101	0.57	1.70	4.28	4.68	3.78	3.06	101.42	4.49
Astragalin	y = 2.17 × 10^4^x + 8.73 × 10^4^	0.9999	0.0353–883	0.01	0.02	3.51	3.92	3.77	3.38	97.79	1.74
Lonicerin	y = 1.11 × 10^3^x + 4.25 × 10^3^	0.9994	22.4–14,000	0.02	0.07	3.45	3.85	4.12	4.41	97.77	3.43
Kaempferol-3-*O*-rutinoside	y = 3.13 × 10^3^x + 9.11 × 10^4^	0.9999	0.632–7900	0.04	0.12	2.31	2.44	3.43	3.62	97.66	1.64
Isoquercitrin	y = 4.33 × 10^3^x + 1.4 × 10^5^	0.9996	2.65–3310	0.20	0.58	1.68	1.54	3.81	2.59	96.34	3.33
Sweroside	y = 34.9x + 1.56 × 10^3^	0.9996	0.841–52,600	0.23	0.69	3.54	3.68	3.09	4.67	96.80	3.12
Secologanic acid	y = 609x + 3.48 × 10^4^	0.9999	1.45–182,000	0.07	0.22	3.02	3.37	3.84	1.08	96.41	2.98
Loganin	y = 3.95x + 1.72 × 10^3^	0.9990	14.1–35,200	4.18	12.54	2.26	2.43	0.37	0.30	98.00	1.84
Secoxyloganin	y = 638x + 1.32 × 10^5^	0.9990	0.189–47,400	0.05	0.16	4.19	4.67	3.16	1.61	98.00	1.70
Loganin acid	y = 1.11 × 10^3^x − 1.98 × 10^5^	0.9993	441.2–22,100	139.27	417.80	2.95	2.54	2.49	1.77	97.33	0.66
Morroniside	y = 10.4x + 8.21 × 10^3^	0.9990	41.1–514,000	7.87	23.60	2.11	1.83	4.69	4.08	97.12	3.21
l-Alanine	y = 1.43 × 10^3^x + 1.78 × 10^4^	0.9997	8.47–10,600	2.23	6.68	3.07	2.60	2.98	4.81	101.09	4.66
l-Serine	y = 351x + 5.93 × 10^4^	0.9997	9.16–11,500	1.34	4.03	2.71	2.54	2.48	4.23	97.96	4.32
l-Proline	y = 1.77 × 10^3^x + 3.04 × 10^4^	0.9996	0.381–23,800	0.09	0.28	2.75	2.86	1.65	3.58	97.09	2.70
l-Valine	y = 6.36 × 10^3^x + 2.17 × 10^5^	0.9995	2.51–1570	0.22	0.64	2.96	3.19	1.40	1.40	97.25	2.09
l-Threonine	y = 1.75 × 10^3^x − 9.1 × 10^4^	0.9996	111–13,900	22.33	66.98	2.83	1.46	0.60	1.35	96.26	2.69
l-Isoleucine	y = 5.15 × 10^3^x + 1.92 × 10^5^	0.9998	2.88–3600	0.42	1.27	0.67	0.63	2.10	1.17	95.31	1.88
l-Leucine	y = 9.64 × 10^3^x + 4.72 × 10^5^	0.9997	3.38–4230	0.71	2.13	1.21	1.28	1.93	0.89	93.89	3.10
l-Aspartic acid	y = 660x + 2.38 × 10^4^	0.9991	4.6–57,500	0.84	2.51	1.49	1.66	1.15	4.09	97.55	2.99
l-Glutamate	y = 1.64 × 10^3^x − 2.82 × 10^5^	0.9998	474–29,600	136.18	408.53	0.69	0.74	2.09	0.93	97.89	1.72
l-Lysine	y = 2.32 × 10^3^x − 2.01 × 10^5^	0.9993	147–12,600	36.78	110.34	0.58	0.65	2.48	0.73	98.52	1.66
l-Histidine	y = 7.11 × 10^3^x + 1.65 × 10^5^	0.9999	59.1–7390	7.66	22.97	1.67	1.45	3.66	1.42	96.14	3.31
l-Phenylalanine	y = 1.7 × 10^4^x + 2.55 × 10^5^	0.9997	25.6–3200	0.55	1.65	1.78	1.28	1.21	0.43	103.21	4.27
l-Arginine	y = 6.54 × 10^3^x + 1.21 × 10^5^	0.9996	0.241–1510	0.04	0.13	1.25	1.19	2.98	1.17	97.99	2.55
Cytidine	y = 4.72 × 10^4^x − 1.94 × 10^4^	0.9995	1.84–230	0.50	1.49	3.39	3.20	1.26	3.93	96.29	3.54
Uridine	y = 861x + 7.0 × 10^3^	0.9997	40.4–5050	7.12	21.37	3.2	3.53	1.31	3.38	98.06	1.71
Adenosine	y = 5.28 × 10^4^x + 3.05 × 10^5^	0.9994	6.31–789	0.95	2.83	1.45	1.41	0.69	0.25	98.61	3.49
Inosine	y = 7.98 × 10^3^x + 2.12 × 10^4^	0.9991	8.78–220	1.85	5.54	1.73	1.87	1.26	2.43	95.89	2.89

**Table 3:** Detailed information of samples.

Species	No.	Batch No.	Habits	Origin
Lonicerae japonicae caulis	S1	180810	Shandong	Ningbo Mingbei traditional Chinese Medicine Co., Ltd.
	S2	20170927	Shandong	Nantong Sanyue Herbal Medicine Co., Ltd.
	S3	20170801	Shandong	Local collection
	S4	171020	Jiangsu	Shanghai medicine holdings Yixing Co., Ltd.
	S5	170501	Shandong	Local collection
	S6	170601	Shandong	Anhui YaoZhiyuan traditional Chinese Medicine decoction Co., Ltd.
	S7	180501	Shandong	Bozhou Beshixin traditional Chinese Medicine slice Co., Ltd.
	S8	1805011	Shandong	Bozhou Beshixin traditional Chinese Medicine slice Co., Ltd.
	S9	18030825	Shandong	Anhui Dichang Pharmaceutical Co., Ltd.
	S10	C16052001	Jiangsu	Zhejiang Yedong Pharmaceutical Co., Ltd.
	S11	180426	Jiangsu	Nantong Sanyue Herbal Medicine Co., Ltd.
	S12	20181104	Shandong	Local collection
	S13	20181105	Shandong	Local collection
	S14	20181103	Shandong	Local collection
	S15	20181102	Shandong	Local collection
	S16	20181101	Shandong	Local collection
Lonicerae japonicae flos	S17	20181108	Shandong	Local collection
	S18	180315	Shandong	Suzhou Boyuan pharmaceutical industry
	S19	2018110506	Henan	Fengqiu
	S20	2018110502	Shandong	Linyi
	S21	180401	Henan	Anhui YaoZhiyuan traditional Chinese Medicine decoction Co., Ltd.
	S22	2018110603	Henan	Fengqiu
	S23	2018110604	Henan	Fengqiu
	S24	2018110303	Hebei	Juluxian Goujijinyinhua market
	S25	C16011901	Henan	Zhejiang Yedong Pharmaceutical Co., Ltd.
	S26	20181109	Henan	Fengqiu
	S27	180701	Shandong	Chongqing Wanli Pharmaceutical Co., Ltd.
	S28	180315	Shandong	Suzhou Boyuan pharmaceutical industry
	S29	2018110601	Henan	Fengqiu
	S30		Henan	Fengqiu
	S31		Henan	Fengqiu
	S32	2018110505	Shandong	Linyi
	S33	2018110302	Hebei	Juluxian Gouqijinyinhua market
	S34	20181103021	Hebei	Juluxian Gouqijinyinhua market
	S35	20181103022	Hebei	Juluxian Gouqijinyinhua market
	S36	2018110301	Hebei	Juluxian Gouqijinyinhua market
	S37	20181103012	Hebei	Juluxian Gouqijinyinhua market
	S38	20181103013	Hebei	Juluxian Gouqijinyinhua market
	S39	2018110504	Shandong	Linyi
	S40	2018110503	Shandong	Linyi
	S41	2018110303	Hebei	Juluxian Gouqijinyinhua market
	S42	20181107	Shandong	Local herbal medicine market
	S43	180607	Shandong	Nantong Sanyue Herbal Medicine Co., Ltd.
	S44	170802	Shandong	Bozhou Beshixin traditional Chinese Medicine slice Co., Ltd.

## Supplementary Materials

Figure S1: Multiple-reaction monitoring (MRM) chromatogram of 47 investigated compounds; Figure S2: The structure of the standard substances; Table S1: Contents of 47 constituents in samples of LJF and LFC.
