# Supplementary material for: Comparison of Multiple Bioactive Constituents in the Flower and the Caulis of Lonicera japonica Based on UFLC-QTRAP-MS/MS Combined with Multivariate Statistical Analysis
Source: Molecules. 2019 May 20;24(10):1936. doi: 10.3390/molecules24101936 (PMC6572465; doi:10.3390/molecules24101936)
Supplement: Supplementary file 1 [file molecules-24-01936-s001.zip › Figure S1.docx]

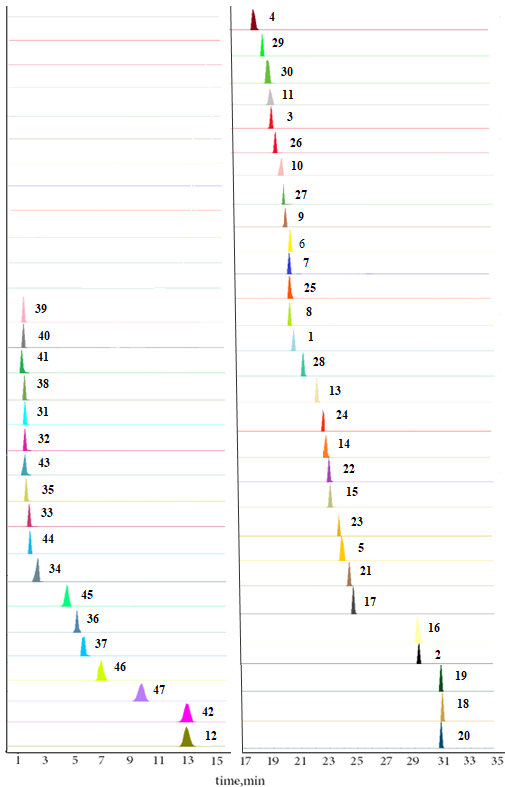


**Figure S1.** Multiple-reaction monitoring (MRM) chromatogram of 47 investigated compounds

Rutin: R1=O-rutinose, R2=OH, R3=OH, R4=H

Isoquercetin: R1=O-glucose, R2=OH, R3=OH, R4=H

Hyperoside: R1=O-galactose, R2=OH, R3=OH, R4=H

Luteoloside: R1=H, R2=glucoside, R3=H, R4=OH

Luteolin: R1=H, R2=OH, R3=OH, R4=H

Lonicerin: R1=H, R2=L-mannose and D-glucose, R3=OH, R4=H

Rhoifolin: R1=H, R2= L-mannose and D-glucose, R3=H, R4=H

Apigenin: R1=H, R2=OH, R3=H, R4=H

Kaempferol-3-rutinoside: R1=O-galactose, R2=OH, R3=H, R4=H

Kaempferol: R1=OH, R2=OH, R3=H, R4=H

Astragalin: R1=glucoside, R2=OH, R3=H, R4=H

Chlorogenic acid: R1=H,R2=Caffeoyl,R3=H,R4=H,R5=H

Neochlorogenic acid: R1=H,R2=H,R3=H, R4=Caffeoyl,R5=H

Cryptochlorogenic acid: R1=H,R2=H,R3=Caffeoyl,R4=H,R5=H

1,3-O-dicaffeoylquinic: R1=H,R2=Caffeoyl,R3=H,R4= Caffeoyl,R5=Caffeoyl

3,5-O-dicaffeoylquinic acid: R1=H,R2= Caffeoyl,R3=H,R4= Caffeoyl,R5=H

3,4-O-dicaffeoylquinic acid: R1=H,R2= Caffeoyl,R3= Caffeoyl,R4=H, R5=H

4,5-O-dicaffeoylquinic acid: R1=H,R2=H,R3= Caffeoyl,R4= Caffeoyl, R5=H

4,5-O-dicaffeoylquinic acid methyl ester: R1=H,R2=H,R3= Caffeoyl,R4= Caffeoyl, R5=CH3

|   Loganin: R=CH_3_  Loganin acid: R=H |   Sweroside: R1=H, R2=Glu, R3=H  Secologanic acid: R1=H, R2=Glu, R3=H |
| --- | --- |

**Figure S2**, The structure of the standard substances.
